# Modeling technology innovation: How science, engineering, and industry methods can combine to generate beneficial socioeconomic impacts

**DOI:** 10.1186/1748-5908-7-44

**Published:** 2012-05-16

**Authors:** Vathsala I Stone, Joseph P Lane

**Affiliations:** 1 Center on Knowledge Translation for Technology Transfer, University at Buffalo (SUNY), New York, NY, USA

## Abstract

**Background:**

Government-sponsored science, technology, and innovation (STI) programs support the socioeconomic aspects of public policies, in addition to expanding the knowledge base. For example, beneficial healthcare services and devices are expected to result from investments in research and development (R&D) programs, which assume a causal link to commercial innovation. Such programs are increasingly held accountable for evidence of impact—that is, innovative goods and services resulting from R&D activity. However, the absence of comprehensive models and metrics skews evidence gathering toward bibliometrics about research outputs (published discoveries), with less focus on transfer metrics about development outputs (patented prototypes) and almost none on econometrics related to production outputs (commercial innovations). This disparity is particularly problematic for the expressed intent of such programs, as most measurable socioeconomic benefits result from the last category of outputs.

**Methods:**

This paper proposes a conceptual framework integrating all three knowledge-generating methods into a logic model, useful for planning, obtaining, and measuring the intended beneficial impacts through the implementation of knowledge in practice. Additionally, the integration of the Context-Input-Process-Product (CIPP) model of evaluation proactively builds relevance into STI policies and programs while sustaining rigor.

**Results:**

The resulting logic model framework explicitly traces the progress of knowledge from inputs, following it through the three knowledge-generating processes and their respective knowledge outputs (discovery, invention, innovation), as it generates the intended socio-beneficial impacts. It is a hybrid model for generating technology-based innovations, where best practices in new product development merge with a widely accepted knowledge-translation approach. Given the emphasis on evidence-based practice in the medical and health fields and “bench to bedside” expectations for knowledge transfer, sponsors and grantees alike should find the model useful for planning, implementing, and evaluating innovation processes.

**Conclusions:**

High-cost/high-risk industries like healthcare require the market deployment of technology-based innovations to improve domestic society in a global economy. An appropriate balance of relevance and rigor in research, development, and production is crucial to optimize the return on public investment in such programs. The technology-innovation process needs a comprehensive operational model to effectively allocate public funds and thereby deliberately and systematically accomplish socioeconomic benefits.

## Background

Achieving societal and economic benefit through evidence-based practice and policy making is an issue that has received increasing attention by social scientists over the last century. The opportunity to influence decisions regarding knowledge implementation with evidence from research, initially pointed out and discussed by Weiss as *research utilization*, has since been discussed under varying terminology, including *knowledge utilizationknowledge transferknowledge dissemination*, and *knowledge exchange*[1,2]. This opportunity is now often called *knowledge translation* (KT) by healthcare fields concerned with implementing research findings to generate positive impacts for patients and professionals (*i.e.*, quality-of-life and efficacious treatment protocols). The challenges to implementing research findings are thought to encompass three aspects: effectively communicating the new knowledge to target audiences in form and content, facilitating its implementation within the user’s context, and tracking and measuring the resulting impacts.

Stakeholders involved in the supply side of scientific research and engineering development outputs (sponsors and grantees) have responded to the challenge of modeling and measuring in different ways. Attempts by the US government to improve the results achieved by government-sponsored research and development (R&D) programs have resulted in various legislative measures, such as the Government Performance and Results Act (GPRA) in 1993, and measurement tools, such as the Program Assessment Rating Tools (PART) by the Office of Management and Budget (OMB) [3]. Note that the revisions in the Government Research and Performance Modernization Act of 2010 establish even more specific performance parameters by more specifically defining a governance structure and by more clearly connecting the underlying plans, programs, and performance information to be reported. Agencies sponsoring R&D activity—and therefore responsible for collecting and reporting evidence of results—have responded by adopting planning and management tools such as the *logic model*[4-7]. In addition, the techniques of research syntheses, meta-analyses, and systematic reviews are assessing the quality of work (performed in terms of rigor and relevance) [8]. These ex-*post facto* techniques measure effects from past studies or assess the validity of their findings to determine their worthiness for future application. By definition, a supply push orientation requires a retrospective orientation because analysis follows completion of the activity. The National Institutes of Health (NIH) catchphrase “bench to bedside” is an explicit statement of such a supply push orientation, where scientific activity is a given, so a focus on their application is a *fait accompli*. The science, technology, and innovation (STI) policies and programs are beginning to consider achieving impact by proactively applying knowledge translation through a demand pull orientation, where the analysis of the problem and the solution precedes the activity—“from bedside to bench and back” [9,10]. Balancing the issues of rigor and relevance becomes even more complex when the government programs that sponsor extramural programs support engineering development activity as well as scientific research activity. Engineering development activity represents a different yet equally rigorous methodology. The difference is critically important for highly regulated and monitored fields such as medical and health technologies [11]. Scientific research methods are designed to generate data analyzed as findings (conceptual discoveries) that can be attributed to the relationship between variables under study and not due to chance. In contrast, engineering development methods are designed to demonstrate that a conceptual relationship between variables can be embodied in a tangible form (prototype invention). The prototype is proof of the principles represented by the conceptual discovery. As the first demonstration of both novelty and feasibility, this prototype can be considered an invention, with the underlying intellectual property entitled to protection under patent laws. However, in order for these outputs from research and development to contribute to socioeconomic benefits, they must be embodied in goods or services exchanged in the commercial marketplace. That is, the invention outputs from engineering development—grounded in the scientific research knowledge base—become inputs to the commercial production process. It is the goods and services produced by industry and deployed in the commercial marketplace that have the capacity to generate social and health benefits to targeted groups, and corresponding economic benefits to a nation [12]. In healthcare, these market innovations are as diverse as medical or assistive devices, pharmaceuticals, and treatment protocols [13].

That said, let’s denote the boundaries of the ensuing analysis. Not all R&D is conducted through grants, nor is all R&D sponsored by governments. However, the substantial investment of public funding with the expressed intent to benefit society warrants a focus on government-operated R&D programs and the innovation-oriented projects that they sponsor.

This paper does not address, and therefore should not be perceived as a criticism of, government-sponsored basic research variously known as fundamental, curiosity-driven, undirected, investigator-inspired, or Mode 1 research. Basic research in the physical, biological, and social sciences is appropriate and contributes directly to the knowledge base in the short term and indirectly to society in unanticipated and often serendipitous ways in the long term. Since serendipity is neither deliberate nor systematic, it is not amenable to advanced planning and therefore outside the boundaries of this paper.

This paper is specifically concerned with those government programs established for the explicit purpose of achieving beneficial socioeconomic impacts through the deliberate and systematic creation and diffusion of technology-based innovations. The allocation of public funding to R&D activities in university, government, and or corporate laboratories is justified by stating that the expected impacts meet national needs that are not being addressed through standard market forces. Many nations operate such technology-based innovation programs. The United States has the National Science Foundation’s Engineering Research Centers (ERCs) [14], Industry/University Cooperative Research Centers (I/UCRC) [15], and Innovation Corps (I-Corps) [16]; National Institutes of Health’s Program on Public Private Partnerships [17]; National Institutes of Standards and Technology’s Technology Innovation Program (TIP) [18]; along with two government agency-wide programs sponsored by the Small Business Administration: Small Business Innovation Research (SBIR); Small Business Technology Transfer (STTR) [19]. Canada funds technology innovation through R&D programs within the Natural Science and Engineering Research Council (NSERC) of Canada, such as the Business-led Network of Centers of Excellence [20], while the European Union jointly funds and coordinates the Research Framework Programme [21] (currently in its seventh 5-year cycle) and the Competitiveness and Innovation Framework Programme. All of these programs support global competitiveness through directed and applied R&D for technology-based innovation. These and others around the globe—including China’s 2050 market-oriented innovation policies [22]—constitute a nontrivial level of public funding invested to deliberately and systematically advance technology-based innovation. All should have process models and performance measures.

In light of the foregoing, this paper presents a conceptual framework for applying KT to the planning, implementation, and evaluation of both research activities and development activities to generate the outputs necessary as inputs to production activities, so industry can generate the socioeconomic impacts desired by society. The focus here is the specific case of translating *technological* outputs (*i.e.*, conceptual discoveries and tangible inventions that are eventually transformed into innovative devices or services in the commercial marketplace). Therefore, the models must accommodate stakeholders beyond the academic community.

A technology-oriented framework defends the argument that, in deriving societal impact from R&D outputs, the issue is not so much how to track and measure the impact as how to plan for and obtain it. Rigor, in both research and development methodologies, is essential to ensuring the credibility of discoveries or verifying the attributes of the prototypes they generate, particularly concerning the efficacy of medical devices or pharmaceuticals. However, relevance is equally indispensable to both methodologies, in terms of relevance to industry as well as to the intended beneficiaries of the innovations. For R&D programs that intend to generate beneficial impacts, the authors contend that relevance is an essential precursor to any project, regardless of whether the project methodology is for scientific research, engineering development, or industrial production.

Evaluation, as a process that investigates the merit and worth of whatever it addresses, plays a crucial but often overlooked role in ensuring both rigor and relevance [23]. The typical R&D project does not engage evaluation systematically, with appropriate emphases on formative and summative forms. This paper argues that the evaluation process can be readily integrated with logic modeling through the Context-Input-Process-Product (CIPP) model developed by Stufflebeam [24-28]. The integration seeks to provide a more comprehensive conceptual framework for R&D project planning, where evaluation explicitly supports both the design of project activities that are relevant as well as a follow-up of their results to impacts. Further, in developing such logic models to represent program theories, a case is made for emphasizing *context evaluations* that link programs with their funded projects. Context evaluations ensure relevance through specific needs assessments at the project level and through broader situation analyses at the program level. To be recognized as a field, implementation science requires such comprehensive frameworks to establish the merit and worth of government-sponsored programs intending to generate socioeconomic impacts through technology-based innovations. This will demonstrate their value to society.

## Methods

### Knowledge translation concepts

A more refined model is needed to address the current under-utilization of outputs from sponsored research and/or development projects. The concept of KT as a solution involves a strategic communication of the knowledge outputs to those interested in using them. A key question is whether knowledge use can be caused by the knowledge producer or if use is determined by the knowledge recipient.

The most commonly used definition of KT, according to the literature, is the one by the Canadian Institutes of Health Research (CIHR). It states the following:

Knowledge translation is a dynamic and iterative process that includes synthesis, dissemination, exchange and ethically sound application of knowledge to improve the health of [citizens], provide more effective health services and products and strengthen the healthcare system [29]. The concept of knowledge translation arose in the context of healthcare, so this definition reflects that context. However, the concept is readily applied to any other field of knowledge creation and application.

Among the many efforts to develop models of KT, the Knowledge-to-Action (KTA) model by Graham and colleagues is notable for its comprehensive inclusion of the aspects involved in communicating knowledge to precipitate action. It incorporates both a Knowledge Creation component and a corresponding Action Cycle component to identify applications and communicate the research-based knowledge to stakeholders [30,31]. The former takes knowledge from the inquiry stage to the tools stage. The latter proposes to identify and address problems relevant to the application of these tools, including the importance of adapting the knowledge to the user’s context.

### Knowledge translation implementation

The KTA model contains two variations: *end-of-grant KT* and *integrated KT*. End-of-grant KT focuses on translating outputs from completed research projects, while integrated KT involves external stakeholders throughout the research project process, from design through to application. Both variations recognize the need to translate the outputs from research projects to eventually demonstrate evidence of output use by stakeholders. In doing so, both restrict the source of knowledge to scientific research methods, the primary actor as a scholarly researcher, and the supporting resources as issuing from a sponsored grant. That is, the KTA model is centered on the professional world of the university scholar. This is appropriate for the majority of research activity, where both sponsors and scholars now seek to extend knowledge use beyond traditional borders.

The KTA model authors recognized the need for yet a third variation that would extend KT beyond scientific research, to encompass the development and production activities required for technology-based innovations. Such an expanded model was needed to address instrumental knowledge use in the creation of devices, pharmaceuticals, and services (personal communications with Dr. Ian Graham). At that time, these downstream knowledge applications were generically represented in the CIHR model of KT as ovals labeled “Contextualization of Knowledge” and “Application of Knowledge” [30]. In this paper, the contents of these generic ovals are described as engineering development and industrial production, respectively.

Appreciating the full potential role for KT in the technology-based innovation process requires one to stand away from the articulated role for academia, in order to view the broader society in which innovation necessarily occurs. Academia relies on a closely aligned network of actors to judge the merit and worth of fellow scholars—hence the term *peer review*. By definition, peers know the traditions, prior literature, and current trajectories for any given topic, and they value knowledge in the form of conceptual discoveries. There is a level of reciprocity involved in mutual progress within a field of study, which includes an expectation that contributions will be cited—that is, the knowledge will be used—by colleagues.

But what happens when those conceptual discoveries from scientific research are offered for uptake and use by stakeholders outside this peer network? Nonacademic stakeholders carry their own value systems on which to judge merit and worth. That is the scenario facing technology-oriented scholars whose own R&D activities are sponsored by government programs that expect to see evidence of downstream application of the scholarly outputs, in the form of prototype inventions arising from engineering development activity, and subsequent commercial goods and services generated through industrial production activity.

Scientific knowledge in the form of conceptual discoveries resides in scholarly literature or in prior applications of that knowledge in practical forms (*e.g.*, existing base of prototype inventions or commercial innovations). When the decision to seek and apply scientific knowledge rests with the target audience, and the decision requires that audience to invest their own time and resources to refine that knowledge for a specific application, then scholars intending to achieve impact must shoulder an additional burden to prove that the knowledge represents value for the decision makers in those target audiences. That burden exceeds the proof required under the original two KTA model variants.

When considering the probability of achieving deliberate and systematic technology-based innovations, the end-of-grant KTA variation is clearly the most risky—a supply push orientation with no assurance that the resulting conceptual discovery is needed, wanted, or even relevant. Even the integrated KTA variation is grounded in the assumption that conducting a research project is a given, and stakeholder engagement will help define the utility and eventual applications. Both KTA variations assume that the knowledge creation begins with some sponsored research activity and ends with the application of the knowledge created.

In contrast, for technology-based innovations, scientific research is only one of three methods involved; research outputs represent knowledge generated in only one of three states, and the question to conduct or not to conduct research is a legitimate one raised by decision makers representing a range of nonacademic stakeholders.

The KTA variation described below as “prior to grant” makes no such assumptions regarding a given and initiating role for scientific research. The opportunity to achieve a technology-based innovation may not require any new conceptual discovery—and therefore no justification to sponsor and conduct scientific research. The state of conceptual science residing in the literature may provide all that is needed, and all may be publicly accessible in the scholarly literature. Similarly, the state of practical engineering may provide all that is needed regarding prototype development, and this too may be publicly accessible in the patent database and industrial literature. If so, there may be little or no need to invest time and money in new prototype inventions.

The authors contend that government programs intending to generate beneficial impacts through technology-based innovations should have the option to sponsor projects that design and implement innovation in response to a validated need, and only then consider what combination of delivery mechanisms and activities to undertake. To this end, Lane & Flagg proposed the third KTA variation called *prior to grant*[12].

Beginning with the end in mind figuratively substitutes a rifle for a shotgun. Articulating and validating a need and then identifying potential solutions permits a program to consider the extent to which the states of science and/or engineering have already generated the knowledge needed to generate the envisioned innovation. That in turn identifies any gaps in the needed knowledge so the sponsor can call for focused scientific research or narrowly defined engineering development activity. The results should maximize the return on investment and facilitate documenting and demonstrating evidence of effectiveness.

### Relevance versus rigor in knowledge creation and adoption

Motivating users to apply technology-oriented knowledge might be a heavier burden on the KT process than is typically assumed, particularly when considering the broader perspective of knowledge use outside of academia and by multiple stakeholder groups. Resistance to change in general or to adopting novel discoveries in particular are concerns that have already been raised in diffusion literature [32]. Beyond that, time and resources available to people or organizations are limited and precious commodities, so any allocation of either is an opportunity cost precluding their allocation to something else. This is the reality of the constant trade-offs required in a fast-paced world besieged by multiple interests competing for support.

A person or organization assessing new knowledge for potential implementation perceives value from its relevance as much as from its rigor. While merit and worth, as proposed by the Joint Committee, are important aspects of knowledge in assessment, new knowledge with high rigor but low relevance is less likely to motivate the effort necessary for adoption [23]. Conversely, new knowledge with high relevance but low rigor may be readily adopted but lack sufficient quality for sustained use in practice.

As noted, these issues are compounded for the use of new technology-oriented knowledge in the context of commercial innovations because the users risk wealth and health, as well as time and opportunity cost. Any KT effort involving the adoption and use of outputs from technology-oriented research and/or development activities might be best served by considering both the merit and the worth at the point of project conceptualization, that is, by identifying the most appropriate methods (rigor) for the activities required, while considering the context and values (relevance) of the target adopters [33]. Fortunately, the earlier-mentioned CIPP Model is up to this task because it emphasizes the target adopter’s context as a critical factor, thus supporting utilization-focused research and/or development as central to new knowledge creation.

From a public policy perspective, a societal context justifies government funding of programs intended to benefit that societal context. The context may be a gap in service provision, a geographic disparity in support, or demographic inequities in access to resources. Government programs address societal issues when the standard market mechanisms lack the necessary economic incentives, but that does not diminish a government’s need to consider those market mechanisms as essential to delivering and sustaining a technology-based innovation underwritten by public funding. Once the issue is framed in terms of societal problems and technology-based solutions, the parity between rigor and relevance becomes self-fulfilling. This reasoning fits the prior-to-grant KT perspective.

### How do technology-oriented programs generate socioeconomic impacts?

Table 1 represents the traditional KT path for the creation and flow of knowledge through multiple methods and stakeholders. The *outputs* from R&D pass through intermediate stakeholders, who cause changes in policies, practices, and products, which are implemented by targeted stakeholders to generate *outcomes* in the short- and mid-term. Such outcomes lead to the desired beneficial *impacts*.

**Table 1:** The path of knowledge translation

				
**Knowledge**	**→**	**Intermediaries**	**→**	**End users**
**Research and development**	**→**	**Intermediate stakeholders**	**→**	**Beneficiaries**
**Immediate results**	**→**	**Short-term/mid-term changes [in user context]**	**→**	**Long-term benefits [to users]**
**Outputs**	**→**	**Outcomes**	**→**	**Impacts**

The unstated implication for technology transfer is that R&D activities culminate in technology-based innovations in the marketplace. That “black box” of innovation can be opened and described in much more detail. In general, the downstream sequence of innovation is the translation of research (R) activity outputs (conceptual discovery) to inputs for development activities, followed by the transfer of development (D) activity outputs (prototype inventions) to inputs for production activities, followed by the deployment of production (P) outputs (finished goods and services) in the market.

### Extending research and development outputs to production impacts

The explicit integration of the three methods of research, development, and production (R-D-P) is necessary for sponsors and managers alike to trace the path of technology-based innovations from concept to impact. Achieving technology-based innovations requires some combination of R-D-P activity, conducted by the appropriate actors, in a reasonably systematic and deliberate fashion. As noted, some of the required R&D activity may have occurred in the past or in a different field of application, in which case the existing outputs need to be identified and transferred into this new application. Some of the relevant prior work may be a serendipitous example of a solution meeting a need, which is a welcome opportunity. However, serendipity is not a basis for policies surrounding systematic and deliberate innovation.

An R-D-P orientation accommodates the dynamic interplay of multiple stakeholders, methods, and outputs/inputs missing from the linear model of innovation, which have also been missing from government STI policies since the 1940s. It was during that time that Dr. Vannevar Bush first suggested that national R&D programs could address important societal needs by connecting basic research to development and application [34]. Despite his intention to coordinate and integrate the sectors and their activity, what survived was an emphasis on basic research, whereby scholars would create a repository of findings from which applied scientists and technology developers could independently draw solutions to problems [35].

Under the linear model of innovation, public agencies fund science-based research, which is assumed to possess sufficient value to spark private investment in the downstream activities necessary to generate socioeconomic benefits. Its dominance over national policies is grounded in an assumed cause-and-effect relationship between the front-end investment in science and the back-end generation of market innovations. The linear model of innovation is often supported or refuted anecdotally, but the critical cause-and-effect link has not been empirically demonstrated as being either reliable or systematic [36,37]. The absence of a proven causal link becomes especially problematic when the sponsored activities do not match the expectations of public policy. For example, government increases the investment in science to improve domestic quality of life or to compete in the global marketplace. Yet, there is a lack of evidence that the investment causes the intended socioeconomic benefits. In the short term, innovation policies expecting outputs from development projects (prototype inventions) or from production projects (commercial goods or service) have difficulty linking these expectations with the prior outputs from research projects (conceptual discoveries). In the long term, the absence of models to track the progress of knowledge through the innovation process works against generating any evidence that might exist.

For policies and projects that intend to precipitate technology-based innovations with socioeconomic benefits, their context includes the full range of R-D-P activities and beyond to their outcomes through stakeholders and, further, to their eventual impacts. This greatly increases the complexity and difficulty of tracking progress from the initial government investments (inputs), through the series of R-D-P activities (inputs/outputs), then on to mid-term outcomes realized by stakeholder applications, and on to the long-term beneficial socioeconomic impacts.

At a minimum, both sponsors and grantees for such innovation programs should align their expectations with society. Doing so would require them to treat the full R-D-P process underlying the introduction of market innovations explicitly in all phases of government-sponsored programs, including (1) requests for proposals, (2) preparation of proposal submissions, (3) proposal reviews, and (4) project management and monitoring. This would be a significant change in the culture and systems through which the public monies are allocated and dispersed, but the gains in documentation and demonstration would be worthwhile. The system would then at least have delivered the envisioned innovations to the stakeholders positioned to deploy and apply them, where use by target audiences would determine the actual impacts and the level of benefit derived.

### The CIPP model to achieve merit and worth in outputs

The fundamental issue underlying accountability in government-sponsored R&D is less about documenting impacts and more about planning for and obtaining such impacts. Effectively planning and implementing research (or development) projects facilitates future tracking and documentation of any contributions made to downstream industrial production and commercialization outcomes, even after the knowledge changes states and when the credit to upstream investigators and sponsors is lost. We reiterate that rigor (merit) and relevance (worth) of knowledge are equally important to any KT effort, particularly those concerning technology-based innovations. The role of evaluation is underappreciated but timely given current demands for demonstrated results.

The CIPP evaluation model proposed by Daniel Stufflebeam [25,27,28,38] is relevant to this discussion because it links evaluation with decision making within systems—such as the technology-based innovation system—and it bears relevance to all of the elements within the knowledge-creation process. Stufflebeam first introduced it in 1966 to guide mandated evaluations of US federally funded projects [27]. The CIPP model shows the enlightening role of evaluation for guiding decisions made in the context of planning for change [24]. The model has since been expanded into a “comprehensive, realistic, practical, and philosophically grounded approach to conducting transparent, defensible, and effective evaluations” [27]. The CIPP model is management-oriented in approach and committed to program improvement and accountability [39].

**Figure 1:**
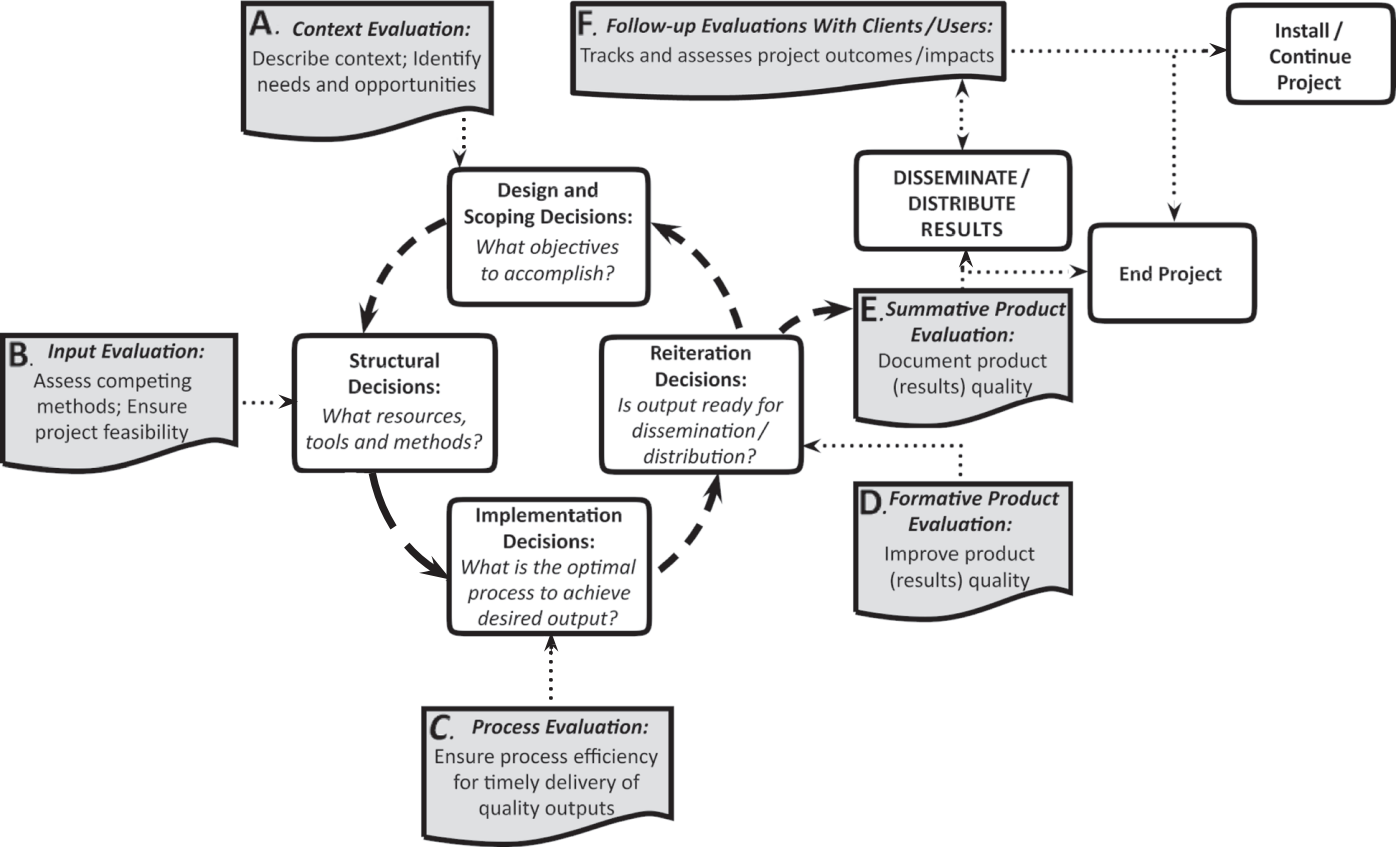
**Evaluation enlightens decision making in a project.** Figure 1 summarizes evaluation's role in guiding a project’s management decision cycle. It is an adaptation of the model proposed by Daniel Stufflebeam [24-28]. The set of four text boxes at the center of the figure represent the flow of the four major decisions through the project cycle: design and scoping decisions on what objectives to accomplish; structural decisions about what resources, tools and methods to use; implementation decisions about the optimal process; and reiteration decisions about the readiness of output for dissemination. They are interconnected by arrows that suggest a circular counter clockwise movement. Corresponding to these four decisions in that order, the supporting evaluation processes are placed in a set of five textboxes A through E, revolving around the central decision boxes. Context evaluation (Box A) identifies and describes context needs & opportunities. Input evaluation (Box B) assesses competing resources to ensure feasible choices; Process Evaluation (Box C) ensures efficiency in delivering quality outputs and on time; Formative product evaluation (Box D) helps improve product quality and Summative product evaluation (Box E) assesses and helps document product quality. Two alternative decisions about the product/results emerge from Box E. The decision to disseminate results is shown through a vertical, upward arrow leading to a box above that suggests results dissemination. This arrow is bidirectional, suggesting repeated dissemination. This connection is interrupted by a horizontal, outward arrow that connects to the alternative decision box that suggests ending the project, a significant post-project possibility. On the other hand, the box of results dissemination connects to Box F placed above, that suggests a follow-up evaluation with product users to assess outcomes and impact of project results. A horizontal, outward arrow from Box F connects to the box that suggests project continuation, another significant post-project decision.

We present a slightly modified version of the CIPP model in Figure 1, where we describe the role of evaluation as applicable to the management and monitoring of any technology-oriented project intending to result in beneficial socioeconomic impacts. The figure shows how evaluative information informs four major decisions of an applied project manager, which are presented in the boxes in the inner circle. The cycle of evaluation is represented by boxes connected to decision boxes: (1) *Context evaluation* (Box A) supplies information about the needs of target beneficiaries, thus guiding project goals; (2) *Input evaluation* (Box B) helps to put the project together through information about the needed and available strategies and resources; (3) when the project process goes into implementation, *Process evaluation* (Box C) helps to find the optimal method for getting the intended output; (4) *Product or output evaluation* (Boxes D and E) assesses the output while it takes shape (formative), and again at the end (summative) to verify that the output meets the intended quality criteria [40,41]. Equally important for program-monitoring purposes given the current emphasis common to all nations, the CIPP evaluation model includes outcome and impact assessments [28].

The CIPP model takes a systemic approach by referring to project goals, inputs, processes and outputs. Needs analysis, which is central to context evaluation, lends direction to the project, taking it closer to the target audience needs, while bringing relevance (worth) to the planned output. Input evaluation ensures that the project is feasible. Process evaluation for research, development, or production methods promotes efficiency and effectiveness. Output evaluation ensures and assesses the quality (merit) of the output and continues to follow up. Thus, evaluation builds relevance from the beginning. It also builds quality by repeated assessment during formative evaluation. Systematic evaluation done according to the CIPP model brings both merit and relevance to the project output.

### The need to knowledge model

**Figure 2:**
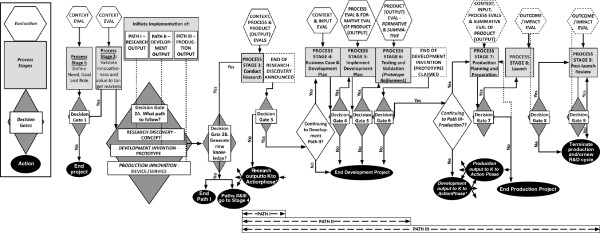
**Evaluation and the R-D-P process.** The flow diagram in Figure 2 explicates the role of evaluation in guiding knowledge generation in the context of technological innovations. The life of the project from beginning to end is shown through a flow of interacting elements from left to right. It represents a stage-gate management model where knowledge outputs from process stages 1 through 9 progress towards the project’s goal achievement in a stage to stage movement, duly controlled by decision gates 1 through 9 at each stage. Data from specific evaluation processes enable the stages to avoid failure at the gates by meeting requirements for outputs. Accordingly, the diagram shows interaction of four types of project elements placed one below the other in four rows. Evaluation processes are shown in nine hexagonal boxes in the top row, held in various combinations of context, input, process, output, outcome and impact evaluations. They connect to nine rectangular boxes in the second row that represent stages 1 to 9 comprising the research to development to production (R-D-P) process of knowledge generation. These are connected to decision gates 1 to 9 represented by nine diamond shaped boxes in the third row. Arrows leaving each gate lead either to the next process stage or to a specific action represented by a corresponding oval box in the fourth row of the diagram. As this row shows, failure to pass through a gate will end the project; and success involves taking knowledge to action. Further, depending on the need for new knowledge (gate 2B), success may imply pursuit of path I (R process) to generate discoveries; or path II (R-D process) to generate inventions or path III (R-D-P process) to generate innovations terminating in product launched and sustained. Or, it may imply a D-P project or a P project.

The CIPP model can guide the research, development, or production phases of project activity to generate and apply knowledge in the required states to achieve innovation. Figure 2 shows the role of evaluation in a project that combines all three methodologies (R, D, and P). Figure 2 is a flowchart representation of the Need to Knowledge (NtK) model, a web-based tool charting the continuum of activities involved in achieving technology-based innovation within a stage/gate structure [42,43]. As the flowchart indicates, several key implications follow from the interaction of evaluation with the process stages and decision gates.

· The R, D, and P processes are performed in stages 1 to 9, where stages 1, 2, and 3 correspond to research activity; stages 4, 5, and 6 to development activity; and stages 7, 8, and 9 to production activity, respectively.

· Problem validation and project goal (solution) definition initiates the R stage in stages 1 and 2. We note that the goal corresponds to the final P output in stage 8, although the P stage continues into stage 9 with assessment and revision.

· Note that evaluation supplies the beneficiary’s (end user) needs well before stage 3. Stages 1 and 2 imply a context evaluation conducted at the level of specificity of a needs analysis for the project. So, the KT process starts *before* conceptualizing any formal R process—a prior-to-grant perspective. Thus, relevance to intended outcomes and impacts is built into the planning of R and/or D, to the extent the project’s plan determines the need to generate new conceptual knowledge beyond what already exists in the published literature.

· Stages 1 and 2 perform the structured data collection and synthesis in preparation for R, while stage 3 performs the empirical research and yields any required conceptual discovery outputs. Stages 4, 5, and 6 prepare for and conduct any needed D leading to tangible prototypes. Stages 7, 8, and 9 apply P methods to transform the prototype into a device or service for the marketplace, commence the product’s launch, and follow its ensuing market performance.

· The flow of knowledge through the R-D-P process is continuous, but the R-D-P activities can also be completed as separate projects. Projects can start at D if the necessary conceptual knowledge from R activity is already available, obviating the need to conduct new research. Or a project can commence at the P phase, if the prior conceptual discoveries are made and the knowledge has been reduced to practice as a functional prototype (*i.e.*, R&D have already been completed). Or, each R, D, or P project can stop at its respective outputs. This is where one often finds a disconnect between the stated intentions of a federally funded program and the project performance of a grantee. A federal program may be funded with the intent to generate innovative devices or services in the marketplace. But grantees may only plan to seek the outcomes for which they are rewarded. For example, scholars may stop at the publication of R outputs to satisfy requirements for tenure and promotion, or inventors may stop at the proof-of-concept prototype necessary to secure patent protection. However, the CIPP model implies that when a plan is comprehensive, the relevance of the final output—the device or service innovation—is integrated from the beginning, during the context evaluation stage, no matter which activity is being conducted in a particular funded project.

Manufacturers are a key stakeholder group in transforming R outputs into D and P outputs. The government and academic sectors do not typically engage in product design, production, distribution, and support. Outputs of projects that intend to generate socioeconomic impacts on a national scale typically pass through the industrial sector. These may be public or private corporations that may operate as for-profit or non-profit entities. The production of goods and services for the marketplace is the domain of industry. It is through industry that technology-based innovations achieve the intended socioeconomic impacts. In this context, society is best served when the government and academia sectors orient their STI programs toward industry as their primary customer.

While a myriad of other groups have a stake in the innovation process, their input is marginal relative to the decisions and commitments of industry. Despite its critical role in the innovation process, industry is not widely embraced by the federally sponsored R&D community, precisely because its critical role is not well represented in the program planning and evaluation models of the government and academic sectors.

### Getting outcomes and impacts from R-D-P project outputs

Evaluation proactively builds relevance into the final output of an R-D-P process by providing evaluative information on beneficiary needs for incorporation into planning. It also guides the intrinsic quality (merit) of output throughout the process, generating formative and summative data useful for program improvement and accountability. Thus, evaluation is an enlightening companion to program planners and managers who set out to obtain R outputs that meet quality standards that are potentially relevant to the ensuing methods of D and P. The ultimate test of relevance of the R-D-P outputs, however, lies in their outcomes (changes achieved through the stakeholders) and their impacts on beneficiaries. The next logical step is to consider how evaluation helps to track evidence of progress beyond the outputs of R or D projects, to assess their actual outcomes through P projects and their eventual socioeconomic impacts.

### Linking the CIPP model to the logic model

Stufflebeam discusses in detail the CIPP model’s explicit guidance in shaping the desired output, which can be applied to R, D, or Production methods. On the other hand, the CIPP model’s guidance in tracking the path of changes beyond the output (*i.e.*, outcomes and impacts) is less explicit—although they are implicit in the perspective of planning for change. Sponsors and investigators engaged in R-D-P projects must follow some structured guidance to assess the extent to which they eventually achieve their intended impacts. The widely accepted process at program level called “logic modeling” serves this purpose because it articulates the structure for linking inputs to the expected outputs, outcomes, and impacts in an *a priori* way. Frechtling refers to the logic model as a tool that describes the theory of change underlying an intervention, product, or policy [44]. An intervention, for example, can be designed to change behavior and thereby solve an identified societal problem. As Rogers points out, logic models can be constructed prospectively for new programs or retrospectively for existing programs [45]. In practice, they have ranged from simple linear versions to nonlinear forms that reflect the complexity of the programs that they represent. They have been used in various settings and by a wide range of public and not-for-profit agencies [46-49].

Evaluators have proposed constructing logic models as a way to describe a program’s theory, or as the causal model that links the intervention with particular results [50]. Logic models are increasingly recognized for their value to both planning and evaluation [51-56]. Some disagree. Stufflebeam and Shinkfield see the value of using a pre-existing theory that is appropriate, but they caution against spending undue time on constructing a model and against considering it as a fixed or validated theory [28]. Scriven and Coryn suggest that program theory construction is neither necessary nor sufficient for conducting sound program evaluation [57]. They point out that it is both expensive and diversionary. However, it is generally recognized that a program theory can explain how the program brings about (or fails to bring about) results, especially if the theory represents an intervention with the cause-effect relations duly identified and described. Among the several uses of a logic model or program theory, Rogers points out that it can provide program staff and other stakeholders a common, motivating vision. It can also help them report a performance story to funders and senior decision makers [45].

**Figure 3:**
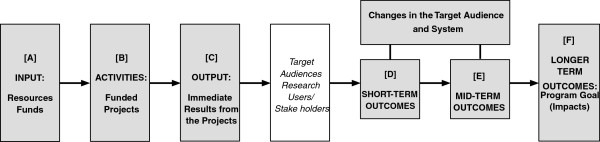
**The logic model of a research and development program: a simple version.** Figure 3 outlines the flow of logic through the operations of a research and development program. It shows a series of 6 text boxes placed next to each other and connected successively by forward arrows that suggest an action chain involving 6 major components of the program. Going from left to right, these components appear as follows: Box A represents the resources and funds that are input to activities shown in Box B, performed under the funded project. Box C represents the output or the immediate results of the program’s projects. Box C then connects to a box that suggests dissemination of results to target stakeholder audiences, or the intended users of research. The dissemination box connects successively to Boxes D and E that represent short-term and mid-term outcomes, respectively, from the disseminated results. These outcomes refer to the changes that take place in the target audiences and in the system that binds them, as described in the elongated box placed above boxes B and C and connected to them. Box E leads to the final text box at the far right which suggests longer term outcomes from the previous flow of effort and represents the program goal in the form of intended impacts.

So what makes logic models appropriate for R&D programs designed to generate technology-based innovations? Figure 3 presents a simple linear version for descriptive purposes. The key elements are the program’s resources (input), activities, and outputs, as well as the program’s short-term, intermediate, and longer-term outcomes [4,5]. When diagrammatically represented, these elements are usually connected by forward arrows to show a logical progression through sequential activities, although this simplified depiction does not obviate the nonlinear and iterative paths taken by components within any one project. In the broader context of technology-based innovation, logic models encompass government sponsorship (input) of R&D projects (activities) generating discoveries or inventions (outputs) to achieve socioeconomic benefits (outcomes).

In Figure 3, the activities (Box B) of the funded project generate the project’s planned output or its immediate results (Box C) using the funding input (Box A). It then disseminates these results to targeted audiences [7]. For technological outputs, these audiences include many categories of stakeholders, including other researchers, manufacturers, practitioners, policy makers/implementers, and technology/information brokers, as well as the end consumers as the beneficiaries from the market innovation [58]. These stakeholders are all potential agents of change in the program’s context, where the changes themselves represent the short-term and mid-term outcomes (Boxes D and E). In fact, any of these stakeholder groups may be sufficient to block progress toward the intended outcome, if their particular interests are not accounted for and served to the extent necessary to gain their support. Ultimately, these outcomes should lead to the program’s broader goal or the desired impact (Box F)—sometimes called the long-term outcome.

As an example of this logical sequence, consider an application by the National Institute for Disability and Rehabilitation Research in its long-range plan for 2005–2009 [49]. Eliminating disparities between people with disabilities and the general population is a long-term area identified in the model. In operational terms, in the context of technology-based R&D, this outcome calls for increased functional independence of consumers with disabilities through the use of assistive technology devices or services. Therefore, actions by manufacturers to introduce new or improved devices or services are viewed as short-term and mid-term outcomes. In turn, the funded R&D projects in the field of rehabilitation engineering are expected to generate the conceptual discoveries and prototype inventions that industry needs to improve upon the existing state of the practice. Boxes A, B, and C describe the project (or program) itself and how it operates, while Boxes D, E, and F describe the effects expected to be caused by them. This causal link is the basis for documenting and demonstrating results.

Related to this specific example, the authors conducted longitudinal case studies on 11 national R&D centers funded in five-year cycles, with the expectation that they would generate technology-based innovations beneficial to persons with disabilities. The case studies revealed that these R&D centers—all affiliated with major US research universities—were less successful at generating technology-oriented outputs, and in achieving external adoption and use by stakeholders, than they had initially estimated in their funded grant proposals. A review of the original proposal narratives showed a marked absence of planning and budgeting for the downstream relationships and activities of development and production. Apparently both the grantees and the reviewers were naïve about the requirements to advance beyond research outputs, and the ensuing grant period was wholly consumed by the research activities in which the grantees were highly trained. A comparison case involving an R&D center experienced in the downstream activities demonstrated a much higher success rate for transfer and commercialization outcomes [58].

These success rates and barriers are similar to those found in analyses of commercialization success by universities in other fields of application [59-61]. To explicitly claim that academic professionals and institutions have the expertise, infrastructure, and incentive systems to independently deliver technology-based innovations benefitting society would sound naïve. Yet that is the implicit claim underlying STI polices. However, this perspective may be shifting to consider how university R&D must be coordinated with downstream stakeholders and activities. According to an analysis by Seigel *et al.*[62], “A key conclusion is that universities and regions must formulate and implement coherent and feasible technology transfer/commercialization strategies.”

### Knowledge translation and logic models

**Figure 4:**
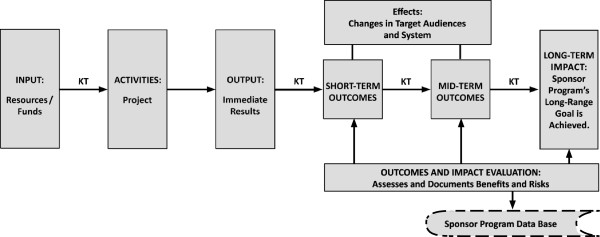
**Implications of KT for the logic model: a simple version.** Figure 4 expands upon Figure 3 by introducing KT or knowledge translation into the outline of operations flowing through a research and development program (or its logic model). It shows a series of six text boxes placed next to each other and connected successively by forward arrows. Going from left to right, these boxes suggest an action chain involving six major components of the program as follows: the first box refers to input or the resources and funds received by the program. The second box refers to activities performed under the funded project, and the third box refers to the output or the immediate results of the program’s projects. This box then connects successively to fourth and fifth boxes that respectively refer to short-term and mid-term outcomes (from the disseminated results). An elongated box placed above these two boxes and connected to them suggests that the outcomes refer to the effects on or changes in the target audiences and the system. The flow of major boxes ends in the sixth text box at the far right which refers to the program goal or the longer term outcomes in the form of intended impacts. A second elongated box is placed below the three boxes of outcomes and refers to outcomes and impact evaluation; this refers to assessing and documenting benefits and risks from the program. The box connects to an oval box below that reads “Sponsor program database” suggesting it is a receptor of evaluation data. The occurrence of knowledge translation in the program operations is shown by placing “KT” adjacent to arrows between appropriate boxes; KT is seen to take place throughout, and is present everywhere except between activities and output.

Figure 4 presents a slight expansion over Figure 3 by adding one additional box. This box represents the role of outcome and impact evaluations, which in turn become inputs to the funding agency’s repository of evidence regarding program outcomes and impacts in the context of their performance accountability. The figure also explicitly inserts the role of KT (note the arrows connecting the boxes) in relation to inputs and outputs. Beyond that, the arrows show the role KT plays in the transition from outputs generated by the project to outcomes generated through the uptake and implementation of these outputs by targeted audiences.

KT represents a more active and tailored approach to communicating new knowledge in any state than the relatively passive approaches of gradual diffusion or scholarly dissemination. KT is also more expansive because the target audiences include multiple stakeholder groups rather than peers within a field of study or practice. The KT arrow connecting the input box to the activities box is a reminder that a KT effort should start even before the conceptualization of the R&D program to ensure optimal relevance to the problem at hand—and thereby to the intended knowledge users. This prior-to-grant perspective is built into the CIPP model through context evaluation, which addresses both program priorities (broader context) and user needs for the project (specific context), and in the logic model’s focus on impact. Combining the two models relates the intended project impacts to the sponsor’s broader program goals, which the authors contend should be the hallmark of any publicly sponsored program intending to benefit society.

## Results

### Integrating CIPP with the logic model to represent technology-based R&D programs

**Figure 5:**
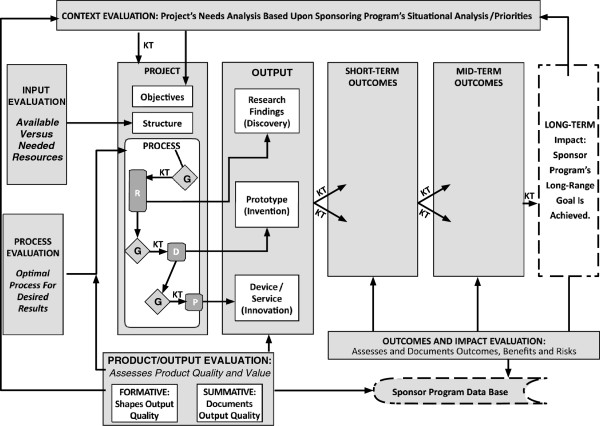
**The bridging role of KT in technology-based R&D.** Figure 5 explicates the bridging action of knowledge translation (KT) in the logic model of a technology-based research and development (R&D) program. The diagram shows six adjacent columns and the model elements in labeled text boxes, placed either inside or around the columns. Arrows connecting boxes and columns suggest the following model dynamics. Connection of context evaluation (box above the columns) to project objectives (second column) suggests the importance of needs analysis and program priorities to knowledge generation. This represents the first KT bridge. While resource assessment from input evaluation (first column) leads to project structure (second column), optimizing procedures though process evaluation (first column) is vital for project process (second column). Interaction within this process suggests a KT path between three distinct processes of research (R), development (D) and production (P) and the stage gate (G); this includes bridges from G to R to G to D to G to P. Outgoing arrows from R, D and P connect these three processes to their respective outputs in the third column: research findings (Discovery), prototype (Invention) and device/service (Innovation). In parallel, the output column also connects to product or output evaluation (box below the columns) which assesses output quality and value, first to improve it (formative evaluation box) and then to document it (summative evaluation box). The output column then leads sequentially to the fourth and fifth columns of short- and mid-term outcomes, and finally to the long-term impact or program goal column. These connections are KT bridges of continued knowledge communication. In parallel, outcomes and impact evaluation (box below the outcome columns) assess column content and send data to the sponsor program database (box below outcome and impact evaluation). This marks the documentation of evidence of project impact, facilitated and solidified by the bridging action of knowledge translation.

**Figure 6:**
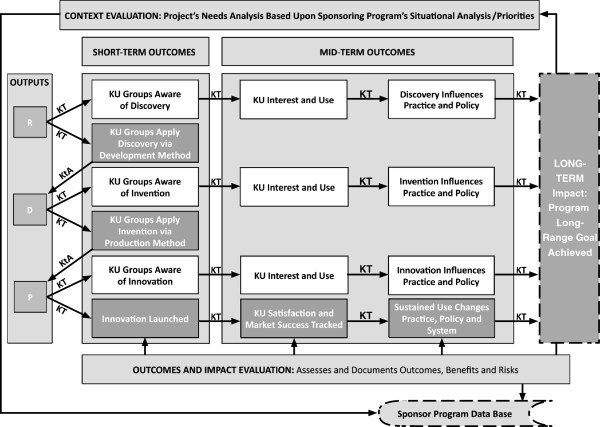
**Progression of R-D-P outputs through outcomes to impact.** Figure 6 summarizes the progression of research (R), development (D) and production (P) outputs of a technology based research and development program towards outcomes and impacts, via knowledge translation (KT). Five adjacent columns sequentially connected by forward arrows show program progress. Arrows leading out of output boxes R, D and P, arranged one below the other in column one, connect to short term outcomes (column two) and represent knowledge translation (KT) to knowledge user (KU) groups. The forked arrows suggest alternate paths of effects generated on the KUs, as represented by alternate rows of boxes across columns. Rows 1, 3 and 5 refer to the first path: awareness of discovery (for R), invention (for D) or innovation(for P) in the short term (column two); interest and use, as well as policy and practice changes in the midterm (columns three and four); and impact in the long term (column 5). Rows 2, 4 and 6 show the alternate effect path. Here the KUs have taken the output to the next stage of technological innovation in the short term- i.e., R to D; D to P; or P to launch. This knowledge-to-action (KtA) effort is represented by 3 return arrows from column two to column one. The resulting path of effects shown in the last row of boxes: innovation launch in the short term; KU satisfaction, tracked market success, and sustained practice and policy changes in the midterm. These effects are more advanced in relation to long term impacts (last column) than the corresponding effects in the first, third and fifth rows. The time to impact is longest in the first row and shortest in the last. It can be verified by the outcomes and impact evaluation (box below columns) and documented through program database (box at bottom of diagram).

**Figure 7:**
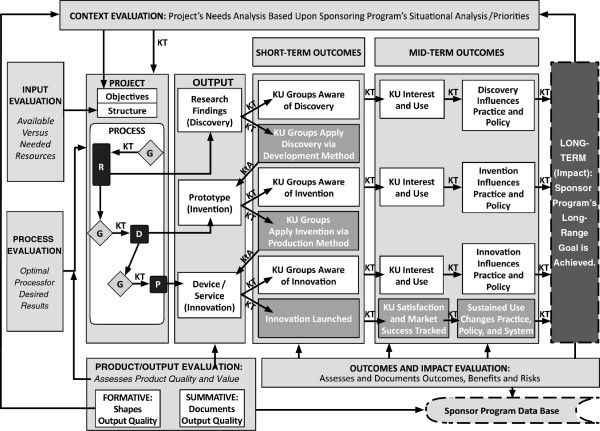
**Planning and evaluating technology-based R&D: role of KT from beginning to end.** Figure 7 presents an overview of planning and evaluating a technology-based research and development (R&D) program, explicitly summarizing the role of knowledge translation (KT) in increasing the likelihood of getting the intended beneficial impacts from its project outputs. It integrates the concepts presented earlier in Figures 5 and 6. It is structured around six columns sequentially connected by arrows suggesting progressive motion. Columns 1 and 2 refer to project activities and project output; the interactions between the column contents and the evaluation activities around the columns were detailed in Figure 5; it showed how knowledge translation (KT) is embedded in those interactions that result in outputs from research (R), development (D) and production (P) processes. Columns 3, 4, 5 and 6 were the primary focus in Figure 6 which presented a detailed view of the KT connections through the progression of outputs (column 2) into long-term impact (column 6), taking two alternate effect paths that cut across short term (column 3), midterm (columns 4 and 5) outcomes. It showed the difference in speed between the two paths in getting impact from an R output: one where knowledge users (KUs) just become aware of the output in the short term and one where they further proceed to take the knowledge to action (KtA) and begin the next process in the technological innovation. Figure 7 captures the above concepts to show the role of KT in effective planning of technology based R&D programs for impacts. The reader is directed to Figures 5 and 6 for details.

Figures 5, 6, and 7 present the results of integrating the CIPP model with the logic model framework. The hybrid model provides technology-based innovation program sponsors and project managers with an operational role for evaluation, from building relevance to tracking impacts.

In Figure 5, the logic model highlights three additional elements:

1. The CIPP evaluation activities are juxtaposed around the project activities box, connected respectively to its objectives, structure, and process.

2. The project activities box is expanded to show R-D-P projects and their relations. Note the KT bridges within the project process and how they link methodological outputs vertically and horizontally. Note also the initial gate before the R phase, where we can avoid the time and expense involved in sponsoring new research if the necessary knowledge already exists in the literature base inside or outside the field of application.

3. The KT bridges go outward from outputs to outcomes; note the forked KT symbols going to outcomes. Here, KT happens in two ways: a general KT and a more focused KT. The first case involves delivering outputs to all stakeholders with potential interest. In the second case, KT may be limited to a specific group, organization, or individual, such as a manufacturer, positioned to treat the knowledge as input to the next method.

Figure 6 then extends the logic model over time. It shows the part of the model where the outputs cause effects in the form of actions by stakeholders (labeled as outcomes). Figure 6 presents the sequence of outcomes resulting from outputs of the R, D, and P activities separately. It also shows two outcome chains in each KT case—general KT versus focused KT. The programs/projects are expected to sequentially obtain outcomes, such as changing the state of stakeholder knowledge from unaware to aware, then on to interest in the knowledge, and eventually to implementation. Implementation should result in changes to practice (*e.g.*, evidence-based applications, prototype construction and testing, commercial device and service manufacturing) or to policy changes (*e.g.*, regulation and reimbursement of devices and services).

The time and effort required to progress through this sequence is partly dependent on the path taken. In Figure 6, one can trace paths of differing length from the output of any method (R, D, or P) to the outcome and impact. The timeframe for research outputs to achieve impacts—particularly for technology-based projects—is extended due to the need to pass through the two downstream methods of development and production. For research projects, achieving impact is likely beyond the scope of funding and beyond the project’s timeframe. This is an important point for project and program accountability. Because accountability requirements only extend to the termination of the funding timeframe, it is not feasible for research projects to demonstrate impact during the award. At best, they can demonstrate the downstream plan through which they or other stakeholders will complete the development and/or production activities and thereby transform outputs into impacts.

Figure 7 combines all of the prior components into a comprehensive diagram to show how the role of evaluation spans the entire innovation process and how KT serves to bridge the components. It shows the links between R, D, and P methods and how they combine to create and deliver a technology-based innovation to the marketplace. It shows the mechanisms involved in generating the socioeconomic benefits expressed in public policies and supported through government programs. Figure 7’s integrated logic model emphasizes the importance of performing context evaluation prior to initiating any efforts intended to generate technology-based innovations. This is an opportune point to apply two forms of context evaluation: (1) project context—the needs and opportunities analysis specific to the project’s immediate context, which provides information necessary for defining project objectives; and (2) program context—the analysis of the broader situational context around the project’s identified problem, which informs funding priorities and request for proposals. Through this approach, the project objectives and the program priorities all become evidence-based.

At the program level, evidence-based information about socioeconomic needs amenable to technology-based innovations helps funding agencies assess grant proposals for relevance, define indicators of impact, and determine how to monitor and evaluate funded projects. The context evaluation ensures that needs remain central to funding priorities and project deliverables.

At the project level, the evidence-based needs analysis aligns the project deliverables with the sponsor’s mission, while ensuring the relevance of project outputs to the intended knowledge users, prior to initiating activities. Figure 7 shows why that approach is preferable to the end-of-grant or integrated approaches to KT for those programs and projects intending to result in beneficial socioeconomic impacts from technology-based innovations [12]. Given the option to do so, why would programs pursue any other path?

Figure 7 integrates key concepts and creates connections to guide construction of integrated logic models for utilization-focused R&D. As a static graphic, it is of necessity simple and linear in form. Yet it can serve as a basis for constructing nonlinear and complex models, as needed, to incorporate and explicate elements that have a bearing on the causal sequence represented by this simple model. It can be readily expanded by individual programs and projects to reflect their unique program characteristics and contexts.

The main point of this integration is to champion the cause of relevance, alongside rigor, through a continuous KT effort that starts at the very beginning of an R&D effort. Future efforts will address how to better integrate government and academic R&D programs supported with public revenues, with privately funded industry efforts to implement the outputs from R&D in technology-based innovations. The outcomes in the commercial marketplace are necessary as incentives for companies to generate the desired socioeconomic benefits. These incentives include revenues from sales paid to corporations to cover their costs (*e.g.*, salaries, materials, and facilities) and profits to owners and shareholders. Of course, a portion of these revenues are paid to the government as taxes (profits to companies, income to employees, taxes on sales), which cycle back through the public coffers to be allocated as public funds used to sponsor R&D. Profitable companies benefit their home nations, so the balance of trade translates directly into national R&D capacity.

## Conclusion

This paper addresses the low level of outcomes and impacts from funded R&D projects, supported by programs expressly intending to generate technology-based innovations with beneficial impacts. This issue is at the heart of accountability for evidence of outcomes and impacts from publicly funded R&D programs. Pointing out that relevance is as important for knowledge utilization as methodological rigor, the paper advocates for starting KT activities at project conceptualization stage when decisions about research and development are open to question, rather than assuming that research (or development) is a required element of any innovation effort. In effect, we are calling for designing utilization-focused R&D, rather than research-driven KT.

The paper argues that government-sponsored technology-oriented R&D projects, intending to generate innovations with socioeconomic benefits, should orient their efforts and direct their outputs toward the commercial marketplace. This venue is where a return on the public investment can be realized through three outcomes: broad and economical diffusion of the innovation to target audiences, revenue from sales captured as profit returned to the innovation producer, and generation of new tax revenue back to the government. The narrative explains that such projects necessarily involve three types of activities (*i.e.*, research, development, and production), each with their own methods and knowledge states. These projects are utilization focused in principle, so the models had to capture key concepts that drive the logic of programs and projects that involve such a comprehensive range of sectors, methods, and activities. The narrative culminated in a conceptual framework that can guide logic-modeling processes for such innovation programs and projects.

The proposed framework integrates elements from the CIPP model of evaluation into the basic, linear logic model format that currently guides program planning and evaluation practice. What links the two models in this framework is the provision of a context evaluation activity prior to initiating any activity. This prior-to-grant perspective elevates the quality of relevance to parity with the quality of rigor—an orientation encompassing the stakeholders who determine success or failure of the entire effort. As a result, funding agencies can focus program goals to ground project objectives in the context of validated needs. The framework also clarifies the roles of process and product evaluations that strengthen the merit and worth of project outputs. The role of outcome evaluations beyond the traditional measures of outputs (*i.e.*, publications, patents) is to assess the actual socioeconomic impacts and deliver those evidence-based results to funders and stakeholders alike.

The next step for expanding the proposed framework in the direction of a well-constructed program theory is an examination of its current simple and linear version. Clearly, the contexts of initiatives for generating technology-based innovations are far from simple and linear. They involve networks of stakeholder interactions that move outputs to outcomes to cause the intended impacts. Often, the interactions are iterative and involve stakeholders in multiple roles (*e.g.*, implementation, managerial, regulatory, evaluative, advisory), so they are not strictly linear. These nonlinear relations should be reflected in an expanded logic model, once the basic concepts and the underlying paradigm are understood and embraced.

Given the possible nonlinear nature of relationships between variables in a technology-based innovation program, it is also useful to think of a systems approach to the creation of logic models. In fact, Rogers and Williams point out the “need to explore systems-based alternatives to the traditional linear logic models used to demonstrate the program theory” [63]. According to them, one way to help people reflect constructively and deeply on the assumptions that underpin the theory as well as the program under study is to incorporate aspects of systems, group dynamics, and learning and cultural theories.

On another level, systems-based thinking may also allow policy makers to appropriately position the logic model within a broader context of the decision-making space in which the funding agency is embedded. For example, it might permit exploration of the model’s link to indicators in the broader system by situating it within the Innovation Systems Framework (ISF) proposed by Jordan, Hage, and Mote [64]. In the ISF framework, the indicators relate to the micro-level, where funds allocation by arena and profile takes place, while analysis of performance by sectors and arena takes place at the meso level of the overall system. Such a systemic approach to the logic model might also explicitly represent and clarify the current distribution of responsibilities regarding accountability within organizations—such as data collection, performance monitoring, and agency oversight—whereby it will address and align the organizational practice with the needs for generating impact-oriented research.

It is clear from the foregoing that building a useful integrated model calls for a joint effort of the evaluator with all the relevant stakeholders involved in the planning of the program. The task calls for dual expertise and clearly is not an either/or proposition. In this sense, the framework proposed in this paper establishes an initial foothold on which a team of planning and evaluation experts could build program theories in their specific contexts of interest.

Finally, efforts to improve society while competing economically necessarily include programs that support technology-based innovations. Quality-of-life issues are paramount in the fields of medicine and healthcare. The process through which scientific knowledge is translated, and technological knowledge is transferred, should be accurately modeled for planning, implementation, and evaluation purposes. Describing the mechanisms underlying technology-based innovations and tracking the indicators of progress are necessary for establishing coherent milestones and accomplishing systematic results. If successful, sponsoring organizations will shift their perspective from the solution-driven “bench to bedside” to the need-driven “bedside to bench and back.”

The appropriate balance of relevance and rigor across all three methods of research, development, and production will help optimize the return on public investment in STI programs. The processes of KT and technology transfer are key to ensuring the progression of knowledge through the various states of conceptual discovery, tangible prototype, and commercial device. A coherent and comprehensive model of the technology-based innovation process is essential for the effective expenditure of public resources through government agencies for the expressed purpose of generating new knowledge with socioeconomic impacts beneficial to society.

### Summary

This paper’s exercise in modeling the technology-innovation process generates five key points:

1. Ensuring awareness and support for—as well as effective implementation of—technology-based outputs from research and/or from development by external stakeholders calls for a targeted and active intervention using KT, rather than a more general and passive dissemination/diffusion effort.

2. The issue of relevance is a critical factor in the decision by stakeholders to adopt and apply knowledge from external sources that involves their commitment to apply internal resources, so it is as important as the issue of rigor during knowledge creation through research and/or development.

3. Prior-to-grant KT with its demand-pull orientation represents a better opportunity to build relevance into innovation-focused R&D programs than do either end-of-grant or integrated models of KT. As a strategy for achieving intended impacts, it is effective for achieving optimal downstream knowledge implementation and efficient for focusing resources and activities on the specified need and the values of the downstream stakeholders responsible for transforming outputs into outcomes and impacts.

4. Both the CIPP model and the logic model can individually serve as planning tools for achieving intended change, but integrating them can provide a more complete framework, particularly for designing and tracking innovation-focused R&D involving a prior-to-grant KT perspective.

5. The integrated framework presented in this paper considers the implementation focus implied in innovation policies and for those R&D programs oriented toward technology-based innovations intended to generate beneficial socioeconomic impacts. It links the underlying key concepts from scientific research, engineering development, and industrial production. The authors intend this generic model format to be widely applicable for the construction of logic models underlying the unique attributes of technology-based innovation programs and their supporting policies.

## Competing interests

The authors declare that they have no competing interests.

## Authors’ contributions

VIS contributed the background on program evaluation and the CIPP model, introduced the logic model as an organizing framework, and integrated the KT and technology transfer processes within it. JPL contributed the background on technology transfer and KT, the rationale for integrating the two within a single framework, the KTA and NtK models, the three states of knowledge, and their collective relevance to technology-based innovations. Both authors read and approved the final manuscript.
